# General Ondansetron Exposure and ICU Outcomes: A Comprehensive Analysis of 85,229 Critically Ill Adults Using the Medical Information Mart for Intensive Care IV (MIMIC-IV)

**DOI:** 10.7759/cureus.96268

**Published:** 2025-11-06

**Authors:** Rohan Muchintala, Shabaaz M Baig

**Affiliations:** 1 Anesthesiology and Perioperative Medicine, Rutgers University New Jersey Medical School, Newark, USA

**Keywords:** critical care, in-hospital mortality, intensive care unit, ondansetron, pharmacotherapy

## Abstract

Introduction

Nausea and vomiting are frequent in critically ill patients and complicate airway safety, medication delivery, and nutrition. Ondansetron is widely used for symptom control, yet its effect on survival in the intensive care unit (ICU) is unclear.

Methods

We conducted a retrospective cohort study using the Medical Information Mart for Intensive Care IV (MIMIC-IV) database. Adult ICU admissions from 2008-2019 were stratified by receipt of ondansetron. The primary outcome was in-hospital mortality; secondary outcomes included ICU length of stay (LOS) and a pragmatic readmission proxy. Analyses included Welch’s t-tests, chi-square tests, multivariable logistic regression, prespecified subgroup analyses, and 1:1 propensity score-matching.

Results

Among 85,229 ICU admissions, 40,205 received ondansetron and 45,024 did not. Mortality was lower with ondansetron than without (9.1% vs 12.9%, p < 0.001). After adjustment, ondansetron was associated with lower odds of death (OR: 0.72, 95% CI: 0.69-0.75). In a 1:1 propensity-matched cohort of 31,221 patients per group, mortality remained lower with ondansetron (11.3% vs 14.6%; OR: 0.74, 95% CI: 0.71-0.78). Ondansetron did not shorten ICU LOS.

Conclusions

Ondansetron exposure during ICU admission was independently associated with reduced in-hospital mortality. These findings suggest a potential survival benefit of a commonly used, well-tolerated antiemetic and support further prospective evaluation of ondansetron as an adjunctive therapy in critical care.

## Introduction

Ondansetron, a selective 5-HT3 receptor antagonist, is one of the most used antiemetics in critical care [1]. Nausea and vomiting are common and clinically significant complications during critical illness, affecting a wide range of ICU patients regardless of their underlying condition [2,3]. These symptoms disrupt multiple aspects of patient care by interfering with the administration of oral medications, delaying the initiation of enteral feeding, and increasing the risk of aspiration [2,3]. Aspiration events are associated with pneumonia, prolonged mechanical ventilation, and increased mortality in the ICU setting [3]. For these reasons, controlling nausea and vomiting is not simply a matter of comfort but is essential for patient safety and recovery.

It has been proven effective across a wide range of causes of nausea and vomiting, including postoperative states, chemotherapy-induced nausea, and gastrointestinal dysfunction associated with critical illness [1]. Ondansetron is available in intravenous and oral formulations, which makes it useful for patients who cannot tolerate oral intake. These characteristics, combined with its long history of use and low cost, have made ondansetron a preferred antiemetic in many ICUs worldwide [1].

Although widely used, ondansetron is not without risks. Regulatory and consensus sources have highlighted the potential for ondansetron to prolong the QT interval in a dose-dependent fashion, which may lead to serious arrhythmias such as torsades de pointes [4-7]. While the absolute risk of these events is low, many critically ill patients have electrolyte abnormalities, organ dysfunction, and concurrent use of other medications that also affect cardiac repolarization [4-7]. These factors increase the likelihood of clinically significant QT prolongation and make ondansetron use more complex in the ICU. Recent studies have demonstrated measurable QT interval changes following typical intravenous doses, supporting the need for thoughtful prescribing and monitoring in high-risk patients [5-7].

In recent years, attention has shifted beyond ondansetron’s role in symptom management to its possible effects on broader clinical outcomes. Several observational studies have reported an association between ondansetron exposure and reduced mortality among certain critically ill groups, such as patients on mechanical ventilation and those with traumatic brain injury [8,9]. Other studies, particularly in perioperative populations, have shown mixed or neutral results, which leaves uncertainty about its true impact on survival and long-term outcomes [10]. These findings highlight the difficulty of interpreting results across different patient populations and study designs.

A recent analysis by Fang and colleagues used the MIMIC-IV database to evaluate early ondansetron use in critically ill adults and found an association between early administration and a decrease in in-hospital mortality, with the strongest effect observed in cardiovascular ICU patients [11]. While these results were important, the study was limited to early exposure and did not evaluate total hospital use or other outcomes such as ICU length of stay or readmissions. It also did not fully address how this association might differ across diverse ICU subgroups. As a result, questions remain about the broader clinical significance of ondansetron in critical care.

The ICU is a valuable setting for answering these questions because high-resolution electronic health record data are available. The Medical Information Mart for Intensive Care IV, MIMIC-IV, is a publicly available de-identified database that contains detailed information on ICU admissions, including medications, vital signs, laboratory data, and outcomes [12]. These data allow researchers to use multivariable modeling and propensity score matching to study treatment outcome relationships with a level of precision that smaller single-center studies cannot provide [12].

There are also potential biological reasons why ondansetron might influence outcomes beyond controlling nausea and vomiting. Serotonin plays a role in gastrointestinal motility, autonomic function, and systemic inflammation [13]. By blocking serotonin signaling through 5-HT3 receptors, ondansetron may reduce inflammation and improve gastrointestinal function, which are both associated with survival in critical illness [13,14]. Improved control of nausea and vomiting may also facilitate early enteral feeding, which has been linked to lower pneumonia rates and better nutritional recovery [2,3,15]. These mechanisms suggest that ondansetron could have indirect benefits that extend beyond its traditional role as an antiemetic.

Despite these possibilities, most studies to date have been limited by small sample sizes, narrow patient populations, and confounding by treatment selection. Methodological challenges in retrospective analyses complicate interpretation. A clear understanding of whether ondansetron use is associated with improved outcomes in a general ICU population is still lacking.

To address these gaps, we conducted a large retrospective cohort study using the MIMIC-IV database to evaluate the association between any in-hospital ondansetron exposure and mortality among critically ill adults. We also examined ICU length of stay and performed prespecified subgroup analyses and propensity score matching to provide a comprehensive evaluation of ondansetron’s potential impact on critical care outcomes. Therefore, this study aimed to evaluate the association between ondansetron exposure and in-hospital mortality among critically ill adults using the MIMIC-IV database, with secondary analyses of ICU length of stay and readmission outcomes.

## Materials and methods

Study design and data source

We conducted a retrospective cohort study using the MIMIC-IV, a publicly available, de-identified critical care database [12]. MIMIC-IV contains detailed, time-stamped information on demographics, admissions, ICU stays, laboratory values, medication orders, and outcomes for patients admitted to the Beth Israel Deaconess Medical Center between 2008 and 2019. Use of the database is approved through a data use agreement, and because the dataset is de-identified, this study was exempt from institutional review board approval.

Cohort definition

We included all adult hospitalizations (≥18 years) recorded in the MIMIC-IV database between 2008 and 2019 that contained at least one ICU stay. For patients with multiple ICU stays during the same hospitalization, only the index ICU stay was analyzed to ensure the independence of observations and avoid double-counting. Encounters were excluded if they were missing any of the following required fields: age, sex, ICU length of stay, discharge status, medication order records, or the readmission proxy variable. This complete case approach ensured a consistent and reliable dataset for regression modeling and propensity score-matching. The final study population included 85,229 hospitalizations, with patients stratified by the presence or absence of ondansetron exposure during their admission. We used complete case analysis to exclude encounters with missing required fields. When hospitalizations included more than one ICU stay, covariates were summarized at the admission level and aligned to the index ICU episode.

Exposure and outcomes

Ondansetron exposure was defined as the presence of any recorded order for ondansetron during the index hospitalization, regardless of route, dose, or timing. This approach was chosen to capture real-world prescribing patterns using the structured medication order data available in MIMIC-IV. While this method does not distinguish between specific administration routes or confirm bedside delivery, it provides a reproducible and consistent way to identify ondansetron use across a large and diverse ICU population. Patients were stratified into two groups based on whether an ondansetron order was present at any point during their hospitalization.

The primary outcome was in-hospital mortality. Secondary outcomes included ICU length of stay and a pragmatic readmission proxy, defined as the presence of more than one hospitalization record in the database. This readmission proxy was included as a covariate in the models rather than as a primary endpoint.

Covariates

Prespecified covariates included age (years), sex, ICU length of stay (days), age ≥ 65 years, ICU stay > 48 h, and the readmission proxy. These factors were selected as clinically relevant variables influencing both treatment decisions and outcomes.

Statistical analysis

Analyses were performed on the complete-case dataset defined in the Cohort Definition. Continuous variables were summarized as mean ± standard deviation and compared with Welch’s t-tests. Categorical variables were summarized as counts and percentages and compared with chi-square tests. For outcomes, we fit univariate and multivariable logistic regression models; effects are reported as odds ratios (ORs) with 95% confidence intervals (CIs), z-statistics, and p-values. All tests were two-sided with α = 0.05.

To address confounding by indication, we estimated a propensity score for ondansetron exposure using logistic regression with prespecified covariates (age, sex, ICU length of stay, age ≥ 65 years, ICU stay > 48 h, and the readmission proxy when available). One-to-one nearest-neighbor matching without replacement was performed on the logit of the propensity score using a caliper of 0.2 times the standard deviation of the logit propensity score. The propensity score was estimated on the complete-case dataset; therefore, no missing values remained in the propensity score model predictors. 

In the unmatched cohort, multivariable models included ondansetron exposure, age, sex, ICU length of stay, and the readmission proxy. In the propensity score-matched cohort, we repeated logistic regression for in-hospital mortality using the matched sample; covariates were retained if they did not introduce collinearity. Model discrimination was assessed using the area under the receiver operating characteristic (ROC) curve, and calibration was evaluated with the Hosmer-Lemeshow test (10 risk deciles).

Subgroup analyses were prespecified by age (< 65 vs ≥ 65 years), sex, and ICU length of stay (> 48 vs ≤ 48 h). Within each subgroup, we reported the adjusted association of ondansetron exposure with in-hospital mortality and the subgroup-specific area under the ROC curve.

All analyses were conducted in Python (version 3.9, Guido van Rossum, Centrum Wiskunde and Informatica (CWI), Amsterdam, the Netherlands) using pandas (Wes McKinney, AQR Capital, Greenwich, CT), SciPy (Travis Oliphant, Rochester, MN), statsmodels (Skipper Seabold and Josef Perktold, statsmodels Developers, Austin, TX), and scikit-learn (Scikit-learn Developers, Inria, Paris, France).

## Results

A total of 85,229 ICU admissions were included, with 40,205 (47.2%) receiving ondansetron and 45,024 (52.8%) not exposed. Patients in the ondansetron group were younger (62.6 ± 16.6 vs 66.6 ± 16.9 years; p < 0.001) and less often male (52.2%, n = 20,973 vs 59.0%, n = 26,548; p < 0.001), had longer ICU stays (3.76 ± 5.67 vs 3.28 ± 4.66 days; p < 0.001), and were less likely to be aged ≥65 years (49.9%, n = 20,054 vs 58.9%, n = 26,506; p < 0.001). Prior admission (readmission proxy) was similar between groups (65.9%, n = 26,482 vs 65.6%, n = 29,549; p = 0.47). In-hospital mortality was lower with ondansetron (9.1%, n = 3,674) than without (12.9%, n = 5,794; p < 0.001). Baseline cohort characteristics are summarized in Table 1.

**Table 1:** Baseline characteristics (ondansetron vs no ondansetron) ICU = intensive care unit; LOS = length of stay. Baseline characteristics of ICU patients stratified by ondansetron exposure. Continuous variables are presented as mean ± SD, and categorical variables are presented as absolute count with corresponding percentage. A p-value of < 0.05 was considered statistically significant.

Variable	Overall	No Ondansetron (n = 45,024)	Ondansetron (n = 40,205)	Test Statistic (t/χ²)	p-value	SMD
Age (Years)	64.72 ± 16.87	66.61 ± 16.88	62.61 ± 16.61	t = -34.81	<0.001	0.239
ICU Length of Stay (Days)	3.51 ± 5.17	3.28 ± 4.66	3.76 ± 5.67	t = 13.54	<0.001	0.094
Male (N, %)	47,521 (55.8%)	26,548 (59.0%)	20,973 (52.2%)	χ² = 397.72	<0.001	0.137
Age ≥65	46,560 (54.6%)	26,506 (58.9%)	20,054 (49.9%)	χ² = 692.42	<0.001	0.181
ICU Length of Stay > 48 h	40,741 (47.8%)	20,388 (45.3%)	20,353 (50.6%)	χ² = 242.56	<0.001	0.107
Readmission Proxy (N, %)	56,031 (65.7%)	29,549 (65.6%)	26,482 (65.9%)	χ² = 0.52	0.469	0.005
In-Hospital Mortality (N, %)	9,468 (11.1%)	5,794 (12.9%)	3,674 (9.1%)	χ² = 298.95	<0.001	0.119

Overall, in-hospital mortality was 11.1% (n = 9,468). In univariate logistic regression, the use of ondansetron was associated with a reduced odds of death (OR 0.681, 95% CI 0.652-0.711; p < 0.001). Age and ICU length of stay were positively associated with mortality, while male sex was not a significant predictor (p = 0.06). Unadjusted associations are shown in Table 2.

**Table 2:** Univariate logistic regression for in-hospital mortality CI = confidence interval; ICU = intensive care unit; LOS = length of stay; OR = odds ratio. Univariate logistic regression for in-hospital mortality. Results are presented as odds ratios with 95% confidence intervals and p-values. A p-value of < 0.05 was considered statistically significant.

Predictor	OR	95% CI	Test Statistic (z)	p-value
Ondansetron Use	0.68	0.652-0.711	-17.22	<0.001
Age (per Year)	1.03	1.025-1.028	35.62	<0.001
Male	0.96	0.920-1.002	-1.86	0.062
ICU Length of Stay (Days)	1.05	1.046-1.053	29.45	<0.001
ICU Length of Stay > 48 h	1.69	1.622-1.769	23.79	<0.001
Age ≥65	1.94	1.854-2.031	28.45	<0.001
Readmission Proxy	0.50	0.481-0.524	-31.36	<0.001

After adjustment for age, sex, ICU length of stay, and prior admission, ondansetron remained independently associated with lower mortality (adjusted OR 0.718, 95% CI 0.686-0.751; p < 0.001). The multivariable model demonstrated moderate discrimination (AUC 0.71) and acceptable calibration (Hosmer-Lemeshow p = 0.24). Fully adjusted results are presented in Table 3.

**Table 3:** Multivariable logistic regression (adjusted odds ratios) aOR = adjusted odds ratio; CI = confidence interval; ICU = intensive care unit; LOS = length of stay. Multivariable logistic regression for in-hospital mortality adjusted for ondansetron exposure, age, sex, ICU length of stay, and the prior admission proxy when available. Results are presented as odds ratios with 95% confidence intervals and p-values. A p-value of < 0.05 was considered statistically significant.

Predictor	Adjusted Odds Ratio (95% CI)	Test Statistic (z)	p-value
Ondansetron Use	0.72 (0.686-0.751)	-14.35	<0.001
Age (Per Year)	1.03 (1.026-1.029)	36.04	<0.001
Male (vs Female)	0.97 (0.926-1.011)	-1.46	0.144
ICU LOS	1.05 (1.050-1.058)	31.54	<0.001
Readmission Proxy	0.48 (0.456-0.498)	-33.01	<0.001

**Figure 1:**
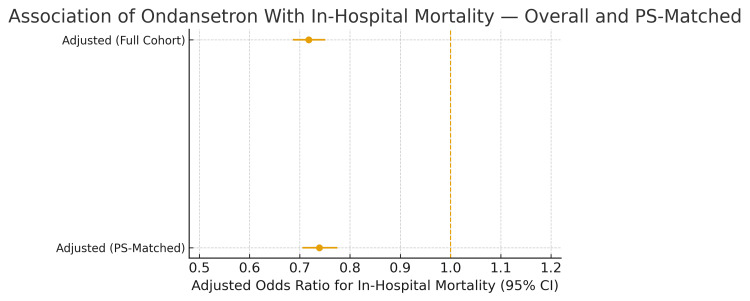
Association of ondansetron with in-hospital mortality in the full cohort and propensity score-matched cohort OR = odds ratio; CI = confidence interval; PS = propensity score. Points show adjusted odds ratios with 95% CIs from multivariable logistic regression models in both the full cohort and the PS-matched cohort.

Figure 1 presents the adjusted associations between ondansetron exposure and in-hospital mortality in both the full cohort and the propensity score-matched cohort. In the full cohort, ondansetron use was independently associated with significantly lower odds of death after adjusting for age, sex, ICU length of stay, and prior admission. This protective association persisted after 1:1 propensity score-matching, demonstrating that the relationship between ondansetron and improved survival was consistent and robust across both analytic approaches.

Prespecified subgroup analyses showed that the association between ondansetron and lower mortality was consistent across strata. Adjusted odds ratios were 0.628 (95% CI: 0.581-0.677; p < 0.001; AUC 0.59) for age <65 years and 0.829 (95% CI: 0.785-0.875; p < 0.001; AUC 0.58) for age ≥65 years; 0.736 (95% CI: 0.693-0.782; p < 0.001; AUC 0.61) for males and 0.765 (95% CI: 0.716-0.817; p < 0.001; AUC 0.62) for females; and 0.703 (95% CI: 0.663-0.744; p < 0.001; AUC 0.59) for ICU length of stay > 48 h. Subgroup results are summarized in Table 4.

**Table 4:** Subgroup analyses (adjusted odds for mortality) AUC = area under the receiver operating characteristic curve; CI = confidence interval; LOS = length of stay; OR = odds ratio. Subgroup analyses for in-hospital mortality stratified by prespecified clinical groups. Results are presented as odds ratios with 95% confidence intervals and p-values. A p-value of < 0.05 was considered statistically significant.

Subgroup	OR	95% CI	p-value	AUC
Age <65	0.63	0.58-0.68	<0.001	0.59
Age ≥65	0.83	0.79-0.88	<0.001	0.58
Male	0.74	0.69-0.78	<0.001	0.61
Female	0.77	0.72-0.82	<0.001	0.62
LOS > 48 h	0.703	0.663-0.744	<0.001	0.59

**Figure 2:**
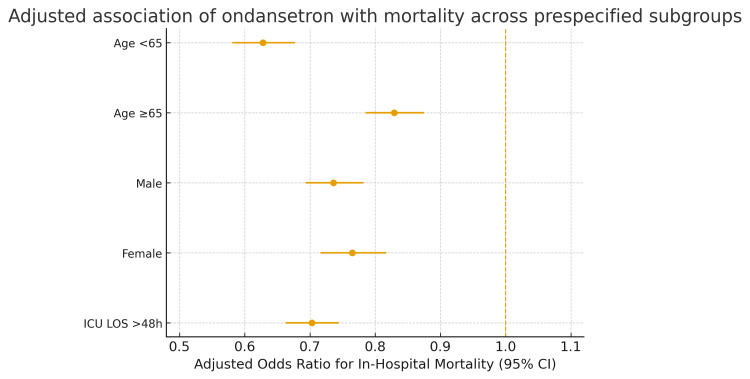
Adjusted association of ondansetron with mortality across prespecified subgroups CI = confidence interval; ICU = intensive care unit; LOS = length of stay; OR = odds ratio. Points show adjusted odds ratios with 95% CIs from multivariable logistic regression models stratified by subgroup.

Figure 2 illustrates the adjusted association between ondansetron exposure and in-hospital mortality across prespecified subgroups. The relationship between ondansetron and improved survival was consistent across different age groups, sex, and prolonged ICU length of stay, demonstrating a uniform protective association.

Before matching, the exposed and unexposed groups differed on several baseline covariates (e.g., age, SMD 0.239; age ≥65 years, SMD 0.128; ICU length of stay, SMD 0.093; male sex, SMD 0.097), reflecting expected treatment-selection patterns. Pre-match covariate balance metrics are shown in Table 5.

**Table 5:** Propensity score balance (before matching) ICU = intensive care unit; LOS = length of stay; SMD = standardized mean difference. Pre-match covariate balance between exposure groups. Values are absolute standardized mean differences for prespecified covariates. An absolute standardized mean difference of less than 0.10 was considered an acceptable balance.

Variable	Overall	No Ondansetron (n = 45,024)	Ondansetron (n = 40,205)	SMD
Age (Years)	64.72 ± 16.87	66.61 ± 16.88	62.61 ± 16.61	0.239
ICU Length of Stay (Days)	3.51 ± 5.17	3.28 ± 4.66	3.76 ± 5.67	0.093
Male Sex	47,521 (55.8%)	26,548 (59.0%)	20,973 (52.2%)	0.097
Age ≥65 Years	46,560 (54.6%)	26,506 (58.9%)	20,054 (49.9%)	0.128
ICU LOS > 48 h	40,741 (47.8%)	20,388 (45.3%)	20,353 (50.6%)	0.076
Prior Admission (Proxy)	56,031 (65.7%)	29,549 (65.6%)	26,482 (65.9%)	0.004
In-Hospital Mortality	9,468 (11.1%)	5,794 (12.9%)	3,674 (9.1%)	0.084

After 1:1 propensity score-matching, 31,221 patients were retained in each group with covariate balance achieved (all standardized mean differences < 0.10). In-hospital mortality was lower with ondansetron (11.3%, n = 3,515) compared to without it (14.6%, n = 4,545; p < 0.001). Matched-cohort mortality and balance are presented in Table 6.

**Table 6:** Propensity score balance (after 1:1 matching) ICU = intensive care unit; LOS = length of stay; SMD = standardized mean difference. Post-match covariate balance in the propensity score-matched cohort. Values are absolute standardized mean differences for prespecified covariates. An absolute standardized mean difference of less than 0.10 was considered an acceptable balance.

Variable	Overall	No Ondansetron (n = 31,221)	Ondansetron (n = 31,221)	SMD
Age (Years)	63.78 ± 16.17	63.51 ± 16.41	64.06 ± 15.93	0.033
ICU Length of Stay (Days)	3.53 ± 5.21	3.29 ± 4.70	3.77 ± 5.65	0.092
Male Sex	41,689 (66.8%)	21,343 (68.4%)	20,346 (65.2%)	0.038
Age ≥65 Years	39,243 (62.8%)	19,388 (62.1%)	19,855 (63.6%)	0.018
ICU LOS > 48 h	35,753 (57.3%)	16,768 (53.7%)	18,985 (60.8%)	0.084
Prior Admission (Proxy)	48,599 (77.8%)	24,077 (77.1%)	24,522 (78.6%)	0.018
In-Hospital Mortality	8,060 (12.9%)	4,545 (14.6%)	3,515 (11.3%)	0.063

After one-to-one matching on the logit propensity score with the prespecified caliper, covariate balance met the predefined threshold. All absolute standardized mean differences were less than 0.10 across the prespecified covariates. Pre-match and post-match standardized mean differences are summarized in Tables 5-6.

In the matched cohort, ondansetron use continued to be associated with reduced mortality (adjusted OR 0.73, 95% CI: 0.705-0.775; p < 0.001); age remained positively associated with mortality, and sex was not significant (p = 0.67). Matched regression results are reported in Table 7.

**Table 7:** Logistic regression in PS-matched cohort CI = confidence interval; OR = odds ratio; PS = propensity score. Logistic regression for in-hospital mortality in the propensity score-matched cohort. Results are presented as odds ratios with 95% confidence intervals and p-values. A p-value of < 0.05 was considered statistically significant.

Predictor	Adjusted Odds Ratio (95% CI)	Test Statistic (z)	p-value
Ondansetron Use	0.73 (0.705-0.775)	-10.21	<0.001
Age (Years)	1.03 (1.024-1.027)	25.29	<0.001
Male (vs Female)	1.02 (0.944-1.038)	0.64	0.670

## Discussion

ICU mortality remains high despite advances in critical care, underscoring the need for therapies that are safe, inexpensive, and widely accessible. In this large retrospective cohort study of more than 85,000 ICU admissions from the MIMIC-IV database, ondansetron exposure during hospitalization was independently associated with a reduced risk of in-hospital mortality. This association remained consistent across multivariable models, prespecified subgroups, and a propensity-matched cohort, suggesting that the observed survival benefit was not explained by baseline imbalances or prescribing patterns. Although ondansetron did not reduce ICU length of stay, this likely reflects survivorship bias rather than a lack of physiologic benefit.

The present findings add to a growing body of literature indicating that ondansetron may confer advantages beyond its established role in controlling nausea and vomiting. Mechanistically, 5-HT3 receptor blockade may attenuate systemic inflammation, modulate autonomic tone, and enhance gastrointestinal motility processes frequently impaired in critical illness and associated with adverse outcomes [13,14,16]. Improved tolerance of enteral nutrition and reduced aspiration risk could further contribute to improved survival [2-4,15]. Together, these pathways offer plausible biological explanations for the observed association between ondansetron use and lower mortality.

Our study expands upon prior investigations by evaluating overall hospital exposure to ondansetron in a large, heterogeneous ICU population rather than focusing solely on early or unit-specific use. Prior analyses, including those examining mechanically ventilated and neurologically injured patients, reported similar mortality reductions [8-10,11]. By confirming this relationship across a broader cohort and analytic frameworks, our results suggest that the potential survival benefit of ondansetron may be generalizable across ICU populations.

From a systems standpoint, ondansetron represents an appealing candidate for quality-improvement initiatives. It is widely available, inexpensive, and familiar to clinicians, with an extensive safety record [1,13]. If future research establishes a causal effect, incorporating ondansetron into ICU care bundles could provide a pragmatic, low-cost means of improving outcomes. In addition to possible mortality benefits, secondary advantages such as decreased aspiration pneumonia and enhanced enteral feeding tolerance could further justify prospective evaluation [2-4,15].

This analysis has several strengths, including its large sample size, high-fidelity dataset, and use of complementary statistical approaches such as multivariable regression and propensity score matching. These methods help mitigate selection bias and enhance confidence in the robustness of the observed associations [12,17]. Nonetheless, limitations remain. Exposure was based on medication orders rather than confirmed administration, and severity indices, such as SOFA or APACHE II, were unavailable. Residual confounding is possible if clinicians avoid ondansetron in patients with QT prolongation risk or organ dysfunction [4-7]. Additionally, immortal-time and collider bias cannot be completely excluded, and the single-center nature of MIMIC-IV may limit external generalizability. Finally, arrhythmia outcomes were not captured, precluding assessment of QT-related adverse events [5-7].

Taken together, the consistency, biological plausibility, and scalability of the findings make ondansetron a promising candidate for further investigation. Prospective, time-aware observational studies and randomized controlled trials are needed to clarify causality, explore dose-response relationships, and evaluate both benefits and potential cardiac risks. If validated, ondansetron could represent a simple, low-cost intervention to improve ICU survival and advance evidence-based critical care practice [1,4,11].

## Conclusions

This large retrospective cohort study found that ondansetron exposure during hospitalization was independently associated with a significant reduction in in-hospital mortality among critically ill adults. Despite its widespread use as an antiemetic, these findings suggest that ondansetron may have benefits that extend beyond symptom control. Given its low cost, favorable safety profile, and familiarity to clinicians, ondansetron represents a promising candidate for further investigation as a potential adjunctive therapy in critical care.

These results highlight the need for future research to clarify the mechanisms underlying this association and to determine whether it reflects a direct therapeutic effect or residual confounding. Prospective studies, including randomized controlled trials, should evaluate whether incorporating ondansetron into ICU care pathways can improve survival and other patient-centered outcomes. Ultimately, confirming these findings could provide a simple, scalable strategy to reduce mortality and improve the quality of care for critically ill patients.
